# EGFR-TKI一线治疗*EGFR*基因突变的晚期非小细胞肺癌临床观察

**DOI:** 10.3779/j.issn.1009-3419.2012.05.09

**Published:** 2012-05-20

**Authors:** 俭杰 李, 莉莉 曲, 星 卫, 红军 高, 伟霞 王, 海峰 秦, 传昊 汤, 万峰 郭, 红 王, 晓晴 刘

**Affiliations:** 1 100071 北京，军事医学科学院附属医院肺部肿瘤科 Department of Pulmonary Neoplasms internal medicine, Affiliated Hospital of Academy of the Military Medical Science, Beijing 100071, China; 2 510080 广州，广州军区机关门诊部内儿科 Pediatrics Department, Command Outpatient Department of Guangzhou Military District, Guangzhou 510080, China

**Keywords:** 肺肿瘤, 表皮生长因子受体, 酪氨酸激酶抑制剂, Lung neoplasms, Epidermal growth factor receptor, Tyrosine kinase inhibitor

## Abstract

**背景与目的:**

研究表明，一线表皮生长因子受体酪氨酸激酶抑制剂（epidermal growth factor receptor tyrosine kinase inhibitor, EGFR-TKI）治疗晚期非小细胞肺癌（non-small cell lung cancer, NSCLC）的客观缓解率及无进展生存期明显优于铂二联的化疗，且耐受性更好。本研究旨在分析EGFR-TKI一线治疗晚期*EGFR*突变阳性的NSCLC患者的疗效与耐受性。

**方法:**

54例晚期NSCLC患者肿瘤标本采用直接测序法证实*EGFR*活化突变（外显子19缺失或外显子21点突变），一线给予EGFR-TKI口服治疗直至疾病进展，观察疗效及不良反应，并进行生存随访。

**结果:**

54例患者外显子19缺失33例（61%），外显子21点突变21例（39%）。均一线接受EGFR-TKI治疗，总体缓解率为90%，中位无进展生存期（progression free survival, PFS）为8.3个月，中位生存期为19.5个月；外显子19缺失患者的中位PFS（9.0个月）较21点突变（7.0个月）时间长（*P*=0.002）。外显子19缺失患者的中位总生存期（overall survival, OS）（25.0个月）较21点突变（16.0个月）时间长（*P*=0.001）；吉非替尼与厄洛替尼疗效相当，但吉非替尼组安全性更好；最常见的不良事件为皮疹和腹泻，有2例患者（4%）出现了3度皮肤毒性反应，2例患者（4%）出现了3度的转氨酶升高，1例患者（1%）出现了3度口腔炎。

**结论:**

存在*EGFR*基因突变的晚期NSCLC患者一线接受EGFR-TKI治疗安全有效，且外显子19缺失比L858R突变疗效更优。

## Abstract (en)

**Background and objective:**

It has been proven that epidermal growth factor receptor-tyrosine kinase inhibitor (EGFR-TKI) significantly benefits advanced non-small cell lung cancer (NSCLC) patients harboring *EGFR* mutations in progression-free survival time with better tolerance. This study is undertaken to analyze efficacy and tolerance of advanced NSCLC patients harboring *EGFR* mutations taking EGFR-TKI as a first-line therapy.

**Methods:**

Tumor samples from 54 patients with advanced NSCLC were examined for *EGFR* activating mutations (deletion mutation in exon 19 and the L858R point mutation in exon 21) by direct sequencing. The patients were first-line treated with oral administration of EGFR-TKI until disease progression. The efficacy and adverse events were observed, and survival was followed up.

**Results:**

Among the patients, 61% (33 of 54) had *EGFR* exon 19 deletion, and 39% (21 of 54) had *EGFR* L858R point mutation. All patients received first-line TKI therapy. The total response rate was 96%, median progression free survival (PFS) was 8.3 months and median survival was 19.5 months. The patients with *EGFR* exon 19 deletion had significantly longer median PFS (9 versus 7 months, *P*=0.002) and longer median overall survival (OS)(25 versus 16 months, *P*=0.001) than patients with *EGFR* L858R point mutation. There is no significance in efficacy between gefitinib and erlotinib, and gefitinib is safer than erlotinib. The most common adverse events were rash and diarrhea. Two (4%) grade 4 skin toxity effects, two (4%) grade 3 aminotransferase level elevations, and one (1) grade 3 stomatitis were observed.

**Conclusion:**

The first-line EGFR-TKI treatment in advanced NSCLC patients harboring *EGFR* mutations is efficient and safe, which is more efficient in patients with *EGFR* exon 19 deletion than those with *EGFR* L858R mutation.

肺癌是当今世界发病率和死亡率最高的恶性肿瘤，其中非小细胞肺癌（non-small cell lung cancer, NSCLC）为最常见类型^[1]^，在靶向药物被批准应用于NSCLC之前，晚期NSCLC标准的一线药物（铂类为基础的化疗）治疗的有效率为20%-30%，而中位生存期仅为10个-12个月^[2]^。靶向药物的异军突起为晚期NSCLC一线治疗带来新的思路，吉非替尼、厄洛替尼以及埃克替尼是目前已上市的表皮生长因子受体酪氨酸激酶抑制剂（epidermal growth factor receptor tyrosine kinase inhibitor, EGFR-TKI）。在回顾性研究^[3-5]^中发现：*EGFR*突变是接受EGFR-TKI类药物治疗晚期NSCLC患者有效率，无进展生存期（progression free survival, PFS）及总生存期的独立预测因素。与疗效相关的突变位点为*EGFR*基因的外显子19缺失或外显子21点突变。

IPASS、FIRST SIGNAL、NEJ002、WJTOG3405等几项随机对照研究^[6-10]^已证实了一线EGFR-TKI治疗晚期NSCLC的客观缓解率及无进展生存期明显优于铂二联的化疗，2009年NCCN指南推荐吉非替尼或厄洛替尼适用于*EGFR*突变阳性的患者。因此，我们应用直接聚合酶链反应（polymerase chain reaction, PCR）测序法，对*EGFR*基因存在外显子19或外显子21突变的54例晚期NSCLC患者，一线给予EGFR-TKI治疗，以了解EGFR-TKI一线治疗晚期NSCLC患者EGFR外显子19或外显子21突变的疗效以及无疾病进展时间。

## 1 材料和方法

### 1.1 研究对象

自2007年6月-2011年12月在本院接受治疗的54例晚期NSCLC患者，中位年龄59.5（30岁-84岁）岁，其中男性14例（26%），女性40例（74%），病理类型：腺癌53例（98%），大细胞肺癌1例（2%）。吸烟状态：吸烟10例（19%）、不吸烟44例（81%）。Ⅲb期4例（7%），Ⅳ期50例（93%）。须具备以下条件：①经细胞学或组织学证实的Ⅲb期或Ⅳ期的未经过治疗的NSCLC；②*EGFR*基因检测外显子19缺失或外显子21 L858R突变；③患者自愿接受EGFR-TKI治疗，并根据其意愿决定其继续或终止治疗，服药前签署知情同意书；④患者具有充足的骨髓功能（中性粒细胞计数 > 1.5×10^9^/L，血小板计数 > 100×10^9^/L，血红蛋白 > 10 g/L）；肝功能（总胆红素 < 1.0倍正常上限，谷丙转氨酶 < 1.5倍正常上限，谷草转氨酶 < 1.5倍正常上限）；肾功能（血清肌酐水平 < 133 mol/L，尿素氮 < 8.3 mol/L）和不吸氧情况下动脉血氧分压 > 60 mmHg（1 mmHg=0.133 kPa）。排除标准包括：未控制的中枢神经系统转移；有严重心肺基础疾病，包括间质性肺病；有习惯性腹泻或便秘等影响药物吸收的胃肠道疾病；必需服用华法令和伊曲康唑等药物；妊娠或哺乳。

### 1.2 *EGFR*基因突变检测方法

应用PCR直接测序法对肿瘤组织或体液标本进行EGFR外显子19、21突变检测^[6]^。

### 1.3 治疗方法

*EGFR*突变阳性晚期NSCLC患者每天口服吉非替尼（250 mg/d）或厄洛替尼（150 mg/d）直至出现不能耐受的毒副反应、病情进展（progressive disease, PD）或死亡。入组前21天内收集病史、体格检查、实验室检查（包括全血细胞计数、尿液分析、肝肾功能）、心电图、胸部CT、腹部B超、头部CT或核磁共振、骨核素扫描做为基线值。入组后每3周进行安全性检查，每6周评价疗效，疗效评判标准按照实体肿瘤疗效评价标准（Response Evaluation Criteria in Solid Tumors, RECIST 1.0）。记录所有的不良事件（CTCAE 3.0）。主要研究终点为无疾病进展时间，次要研究终点为客观有效率、生存期和安全性。自患者接受治疗开始随访，因PD终止治疗者仍进行随访。

### 1.4 统计分析

所有统计分析均采用SPSS 13.0软件包。中位生存期和中位PFS采用*Kaplan-Meier*方法计算。无进展生存期定义为无肿瘤进展或死亡的生存期，从口服EGFR-TKI治疗开始计算，直至首次观察到肿瘤进展。生存期从EGFR-TKI治疗开始计算，直至死亡或最后一次门诊随访。使用卡方检验分析EGFR状态、临床特征和肿瘤对EGFR-TKI治疗反应间的相关性。

## 2 结果

### 2.1 患者特点

本研究共纳入了自2007年6月-2011年12月在本院接受治疗的54例晚期NSCLC患者，所有患者的肿瘤组织或体液标本经直接测序法检出存在EGFR外显子19、21突变，给予一线口服吉非替尼（250 mg/d）或厄洛替尼（150 mg/d）。表 1显示了54例接受EGFR-TKI治疗的患者特征，其中中位年龄为59.5岁，大多数患者为腺癌，从不吸烟的女性，其中ECOG体能状态0分-1分37例（69%），≥2分17例（31%），吸烟或曾吸烟患者10例（19%），其中基因突变与性别、病理类型和吸烟状况有统计学差异（*P*值均 < 0.001）（表 1）。

**1:** 基线特征 Characteristics of all patients

Characteristics	*n*=54
Age	
Median (yr)	59.5 (30-84)
< 65 (%)	32 (59%)
≥65 (%)	22 (41%)
Gender	
Male (%)	14 (26%)
Female (%)	40 (74%)
Tumor type	
Adenocarcenoma (%)	53 (98%)
Non-characteristics (%)	1 (2%)
Smoking status	
Smoking (%)	10 (19%)
Non-smoking (%)	44 (81%)
*EGFR* mutation	
Del19 (%)	33 (61%)
L858R (%)	21 (39%)
ECOG PS	
0-1 (%)	37 (69%)
≥2 (%)	17 (31%)
Tumor stage	
Ⅲb (%)	4 (7%)
Ⅳ (%)	50 (93%)
ECOG: Eeastern Cooperative Oncology Group; PS: performance status.	

### 2.2 基因突变分析

54例患者中44例检测标本来源于新鲜肿瘤组织或石蜡切片，6例来源于胸水，有4例患者同时从组织及胸水标本中检测出突变，且组织与胸水突变结果一致。33份肿瘤标本被检出存在外显子19突变，占突变总数的61%，1例（2%）EGFR外显子19 P753L突变；21份肿瘤标本被检出外显子21点突变，占突变总数的39%；检测中还发现了1例EGFR外显子21 L861Q突变（2%），L861P突变1例（2%）。

### 2.3 疗效反应

在54例可评估患者中，接受吉非替尼治疗的患者26例，厄洛替尼治疗的患者28例，没有完全缓解的病例，其中部分缓解49例（90%），疾病稳定3例（6%），疾病进展2例（4%），总体缓解率为90%，疾病控制率96%。我们还发现较好的疗效反应与外显子19缺失突变而非L858R突变相关，外显子19缺失患者的疾病控制率100%，高于外显子21点突变90%（*P*值< 0.05），疾病进展2例为外显子21点突变。

### 2.4 无进展生存期和总生存期

截止2012年1月，共有38例患者死亡，1例因脾囊肿出血中断治疗，3例患者失访。中位PFS为8.3个月，中位生存期为19.5个月。1年生存率为63%，47例患者已出现疾病进展或死亡。外显子19缺失患者的中位PFS（9.0个月）较外显子21点突变PFS（7.0个月）时间长（*P*=0.002）。外显子19缺失患者的中位总生存期（overall survival, OS）（25.0个月）较外显子21点突变OS（16.0个月）时间长（*P*=0.001）（图 1）。当按照体能状态、年龄及吸烟类型进行分析时，无进展生存期没有统计学差异。

**1:**
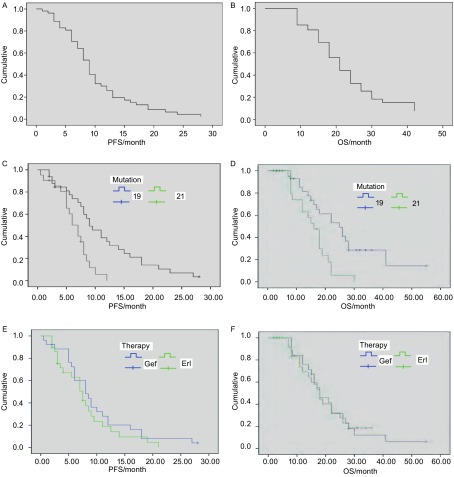
患者的无进展生存及总生存*Kaplan-Meier*生存曲线。A：所有患者的PFS生存曲线；B：所有患者的OS生存曲线；C：外显子19与外显子21突变患者的PFS生存曲线；D：外显子19与外显子21突变患者的OS生存曲线；E：口服吉非替尼与厄洛替尼患者的PFS生存曲线；F：口服吉非替尼与厄洛替尼患者的OS生存曲线。 *Kaplan-Meier* curves of PFS and OSl. A: PFS in all patients; B: OS in all patients; C: PFS comparing between 19 exon mutation and 21 exon mutation; D: OS comparing between 19 exon mutation and 21 exon mutation; E: PFS comparing between gefitinib and erlotinib; F: OS comparing between gefitinib and erlotinib. PFS: progression free survival; OS: overall survival.

### 2.5 吉非替尼与厄洛替尼比较

虽然是回顾性分析，但接受吉非替尼治疗的患者26例，厄洛替尼治疗的患者28例，故从基线特征（表 2）、疗效及不良反应做了比较，在临床选择口服吉非替尼或厄洛替尼时，我们发现女性、非吸烟患者多口服吉非替尼，而吸烟、男性患者更倾向于厄洛替尼（*P*值分别为0.008和0.021）。也对吉非替尼及厄洛替尼疗效做了分析，出现病情进展2例患者口服吉非替尼，病情稳定3例患者口服厄洛替尼，总体缓解率没有差异。二者的PFS（*P*=0.282）及OS（*P*=0.944）均无统计学差异。在不良反应方面，吉非替尼总体低于厄洛替尼，吉非替尼皮疹8例（30%），腹泻4例（15%）而厄洛替尼皮疹23例（82%），腹泻9例（32%），吉非替尼出现3度以上不良事件为1例（3度转氨酶升高），其余4例3度不良反应均发生在厄洛替尼组。

**2:** 吉非替尼与厄洛替尼患者特征比较 Characteristics comparing between gefitinib and erlotinib

Characteristics (*n*=54)	Gefitinib (*n*=26)	Erlotinib (*n*=28)	*P*
Male/Female	3/23	11/17	0.021
Smoking/non-smoking	1/25	9/19	0.008
Adenocarcenoma /Non-adenocarcenoma	26/0	27/1	0.510
19 del/L858R	14/12	19/9	0.219

### 2.6 总体不良反应

本研究对所有患者的不良反应进行评价。最常见的不良事件是皮疹31例（57%）、腹泻13例（24%）、食欲下降12例（22%）和口腔炎5例（9%）。有2例患者（4%）出现了3度皮肤毒性反应，2例患者（4%）出现了3度的转氨酶升高，1例患者（1%）出现了3度口腔炎，1例患者因脾囊肿出血中断治疗。出现3度以上不良事件的患者均停药减量治疗，但未影响到效疗，且减量后短时间内副反应恢复至1度继续服药（表 3）。

**3:** 主要的不良反应（≥3%） Main side effects (≥3%)

Side effects	Grade (*n*=54)	
	Any grade	Grade 3-4
Rash (%)	31 (57%)	2 (4%)
Diarrhoea (%)	13 (24%)	0
Appetite loss (%)	12 (22%)	0
High ALT (%)	5 (9%)	2 (4%)
Nausea, vomit (%)	1 (2%)	0
Paronychia (%)	2 (4%)	0
Stomatitis (%)	5 (9%)	1 (2%)
Fatigue (%)	1 (2%)	0

## 3 讨论

本研究纳入的54例晚期NSCLC患者均检测到存在*EGFR*基因敏感突变，突变更常见于腺癌（98%）、从不吸烟的女性（74%），有1例大细胞肺癌出现19缺失突变，其中吸烟或曾吸烟患者10例（19%），较OPTIMAL^[6]^研究报道的突变患者吸烟比率（28%）及其它相关研究结果^[1, 9]^（约30%）为低，可能与病例数较少有关。其中基因突变与性别、病理类型和吸烟状况有统计学差异（*P*值均 < 0.001）。

在所有可评估患者中，接受EGFR-TKI治疗的总体缓解率为90%，与回顾性研究和前瞻性研究报告^[6-10]^的吉非替尼及厄洛替尼总体缓解率接近，其中OPTIMAL为83%，EURTAC研究为71%，IPASS研究为71%，WJTOG3405研究及NEJ002研究分别为62%及74%，更高的有效率可能与外显子19缺失突变较多相关。

疗效分析显示，54例患者一线接受TKI治疗的中位无进展生存期为8.3个月，中位生存期为19.5个月，相较于既往报道^[2]^的铂类联合三代新药方案一线化疗通常得到的30%的有效率、5个月的中位无进展生存期和12个月的中位生存期有提高。而且这些结果与EGFR-TKI一线治疗NSCLC患者的其它随机研究结果一致^[6-10]^，在先前无吸烟史或曾少量吸烟的肺腺癌患者中进行的IPASS结果显示：吉非替尼的获益仅限于伴有*EGFR*突变的患者，中位PFS为9.5个月，在WJTOG3405及NEJ002研究中，EGFR-TKI一线治疗*EGFR*突变阳性NSCLC患者中位PFS分别为9.2个月及10.4个月，而EURTAC研究及OPTIMAL研究EGFR-TKI一线治疗突变阳性患者分别获得9.7个月及14个月的无进展生存期。

*EGFR*基因突变的动力学分析显示：外显子19缺失比L858R突变对厄洛替尼抑制更敏感，此结果已被回顾性研究证实^[3-5, 11, 12]^，即EGFR-TKI治疗EGFR外显子19缺失的患者疗效较L858R突变的患者好，我们也对外显子19及21突变患者的疗效做了比较，本研究纳入的54例患者中，EGFR外显子19缺失突变的发生率（61%）高于EGFR外显子21点突变的发生率（39%）（*P* < 0.001）。且外显子19缺失患者的中位PFS（9.0个月）较外显子21点突变PFS（7.0个月）时间长（*P*=0.002）。外显子19缺失患者的中位OS（25.0个月）较外显子21点突变（16.0个月）时间长（*P*=0.001）。最近西班牙开展的一项关于厄洛替尼的前瞻性研究对突变类型及无进展生存期进行分析^[13]^，虽然在统计学上无明显差异（*P*=0.02），但有外显子19缺失的患者接受EGFR-TKI治疗较L858R突变的患者疗效好的趋势。

虽然是回顾性分析，我们发现口服吉非替尼与厄洛替尼患者相当，故对两组患者基本特征、疗效及不良反应做了比较，对于男性吸烟患者，临床医生在选择EGFR-TKI治疗时，更倾向于厄洛替尼。吉非替尼组及厄洛替尼组无论是总体缓解率，还是PFS及OS均未发现统计学差异。但吉非替尼组的不良反应发生率低于厄洛替尼组。2009年世界肺癌大会上一项来自韩国的前瞻性随机Ⅱ期研究直接比较了吉非替尼与厄洛替尼二线治疗晚期非小细胞肺癌患者的疗效，研究结果显示，两组的客观缓解率与疾病控制率相当，而PFS及OS未发现统计学差异，且吉非替尼的安全性更好。随后分别发表于2010年Cancer上的3项直接比较易瑞沙和厄洛替尼的回顾性研究也得出了同样的结论^[14]^。由于我们的数据源于回顾性分析，且研究者在选择患者方面有倾向性，而已知的回顾性与前瞻性研究证据尚不充分，仍需更多的吉非替尼与厄洛替尼头对头、前瞻性、随机对照研究来比较二者的疗效及安全性。

所有接受吉非替尼及厄洛替尼治疗发生的皮肤毒性反应或腹泻大多数能够根据现有指南^[15-17]^得以控制，不需要减量治疗，而3度以上不良事件的患者通过停药减量治疗，均未影响到效疗，且减量后短时间内副反应恢复至1度继续服药。

综上所述，对于存在*EGFR*基因突变的晚期NSCLC患者，一线接受EGFR-TKI治疗能够获得更高的缓解率以及更长的无进展生存期，且副反应轻，患者有着更好的生活质量，外显子19缺失比L858R突变疗效更优。因此对于女性、从不吸烟者和非鳞状细胞癌的晚期NSCLC患者进行*EGFR*基因突变的检测是必要的，一线突变阳性患者选择EGFR-TKI治疗是可行的。
